# Classification and hemodynamic characteristics of delayed intracerebral hemorrhage following stent-assisted coil embolism in unruptured intracranial aneurysms

**DOI:** 10.3389/fneur.2024.1268433

**Published:** 2024-02-19

**Authors:** Zeng-Bao Wu, Xue-Yan Wan, Ming-Hui Zhou, Yan-Chao Liu, Ali Abdi Maalim, Zhuang-Zhuang Miao, Xiao Guo, Ying Zeng, Pu Liao, Li-Ping Gao, Jian-Ping Xiang, Hua-Qiu Zhang, Kai Shu, Ting Lei, Ming-Xin Zhu

**Affiliations:** ^1^Department of Neurosurgery, Tongji Hospital, Tongji Medical College, Huazhong University of Science and Technology, Wuhan, China; ^2^Department of Pathology, Union Hospital, Tongji Medical College, Huazhong University of Science and Technology, Wuhan, China; ^3^ArteryFlow Technology Co., Ltd., Hangzhou, China

**Keywords:** delayed intracerebral hemorrhage, hemodynamics, stent-assisted coil embolization, intracranial aneurysms, endovascular treatment

## Abstract

**Background and objective:**

Stent-assisted coil (SAC) embolization is a commonly used endovascular treatment for unruptured intracranial aneurysms (UIAs) but can be associated with symptomatic delayed intracerebral hemorrhage (DICH). Our study aimed to investigate the hemodynamic risk factors contributing to DICH following SAC embolization and to establish a classification for DICH predicated on hemodynamic profiles.

**Methods:**

This retrospective study included patients with UIAs located in the internal carotid artery (ICA) treated with SAC embolization at our institution from January 2021 to January 2022. We focused on eight patients who developed postoperative DICH and matched them with sixteen control patients without DICH. Using computational fluid dynamics, we evaluated the hemodynamic changes in distal arteries [terminal ICA, the anterior cerebral artery (ACA), and middle cerebral artery (MCA)] pre-and post-embolization. We distinguished DICH-related arteries from unrelated ones (ACA or MCA) and compared their hemodynamic alterations. An imbalance index, quantifying the differential in flow velocity changes between ACA and MCA post-embolization, was employed to gauge the flow distribution in distal arteries was used to assess distal arterial flow distribution.

**Results:**

We identified two types of DICH based on postoperative flow alterations. In type 1, there was a significant lower in the mean velocity increase rate of the DICH-related artery compared to the unrelated artery (−47.25 ± 3.88% vs. 42.85 ± 3.03%; *p* < 0.001), whereas, in type 2, there was a notable higher (110.58 ± 9.42% vs. 17.60 ± 4.69%; *p* < 0.001). Both DICH types demonstrated a higher imbalance index than the control group, suggesting an association between altered distal arterial blood flow distribution and DICH occurrence.

**Conclusion:**

DICH in SAC-treated UIAs can manifest as either a lower (type 1) or higher (type 2) in the rate of velocity in DICH-related arteries. An imbalance in distal arterial blood flow distribution appears to be a significant factor in DICH development.

## Introduction

Symptomatic delayed intracerebral hemorrhage (DICH) emerges as a grave postoperative complication following flow diverter (FD) interventions for intracranial aneurysms, characteristically manifesting hours to days subsequent to the procedure, with intracerebral hemorrhage (ICH) occurring in the distal area of the parent artery, precipitating gradual neurological decline (1, 2). However, our study found that DICH can also afflict patients with unruptured UIAs undergoing stent-assisted coil (SAC) embolization. Presently, the causes and pathogenesis of this life-threatening complication remain elusive, with prediction yet unattainable (3–5). Various pathophysiological conjectures have been posited for DICH, with dual antiplatelet therapy (DATP) being a predominant factor (2, 24, 25), alongside hemorrhagic transformation of cerebral infarctions (2, 6, 7), occlusive embolic events (22), and hemodynamic shifts (1, 4, 5, 22–23). Specifically, the hemorrhagic risk associated with SAC for UIAs under DATP is documented between 2.2% and 2.6% (28, 26), and the hyper-responsiveness of clopidogrel for antiplatelet effects significantly increases the risk of bleeding in endovascular procedures (10, 8). Hemodynamic modifications in the distal parent artery post-FD are also implicated in DICH pathogenesis (1, 5, 12–14), yet their occurrence post-SAC remains unstudied. Employing computational fluid dynamics (CFD), our study probes the hemodynamic alterations in DICH-related artery of the distal branch post-SAC treatment, seeking insights into the underlying mechanism. Novel to our research is the classification of DICH based on hemodynamic changes and blood flow distribution discrepancies in SAC-treated distal arteries. Our findings aim to bolster preventative strategies against potentially fatal complications in UIAs managed with SAC treatment.

## Materials and methods

### Patients

This study received approval from the institutional medical ethics committee of Tongji Hospital, which is associated with Tongji Medical College of Huazhong University of Science and Technology. A retrospective analysis of the medical records database was conducted for patients diagnosed with UIAs and treated with SAC embolism at our institution between January 2021 and January 2022.

### Patients selection adhered to these criteria

(1) UIAs situated within the internal carotid artery (ICA); (2) Post-SAC DICH confirmation via computed tomography (CT); (3) Technical success of each operative procedure; (4) High-resolution three-dimensional digital subtraction angiography (3D-DSA) suitable for CFD simulation.

Cases where DICH was a consequence of interventional maneuvers, such as microwire perforation or stent displacement, were omitted. Exclusions also included patients with DICH not definitively attributable to the ACA or MCA territories.

From a total of 619 patients with 661 UIAs undergoing SCA treatment, nine patients (1.5%) experienced unanticipated DICH complications. One case, involving an MCA bifurcation aneurysm, was omitted. The resultant eight patients with ICA aneurysms constituted the DICH study group. These individuals underwent SAC embolism, with six using Enterprise 2 (EP 2) stent (Codman Neurovascular, Raynham, Massachusetts, United States) and two the Neuroform EZ stent (Stryker Neurovascular, Fremont, California, United States).

For each DICH case, we identified two control patients lacking DICH manifestations. These controls, observed over six months, exhibited no angiographic or radiographic signs of DICH. Matching prioritized aneurysm location, size, and stent type (either EP 2 stent or Neuroform EZ stent with coils), while secondary factors included age and gender. Ultimately, sixteen control patients were perfectly matched.

We compiled demographic and clinical data, encompassing gender, age, hypertension history, perioperative blood pressure, and platelet inhibition. Additional data covered aneurysm dimensions, neck width, and stent variety. We documented initial angiographic outcomes, the timing and location of hemorrhagic events, and detailed treatment interventions, including any craniotomy performed.

The modified Roy-Raymond classification (MRRC) assessed angiographic outcomes, categorizing them as (15): Class I (complete obliteration), Class II (residual neck), and Class III (partial occlusion).

### Computational modeling and hemodynamic simulations

Patient-specific aneurysm reconstructions was derived from 3D original sectional images, utilizing Geomagic Studio version 12.0 software (Geomagic, Research Triangle Park, North Carolina, Unites States) for segmentation and model refinement. The method of virtual implantation for stent and coil established in the previous research was adopted in this study (16). All stents including Enterprise 2 and Neuroform EZ were modeled using NX 12.0 (Siemens PLM Software, Plano, Texas, United States), and imported into ABAQUS version 6.14 (SIMULIA, Providence, Rhode Island, United States) to simulate the clinical implantation. Generation of coils was performed in MATLAB (MathWorks, Natwick, Massachusetts, United States). The implantation of stents and coils was carried out in stages following the actual order in clinical practice. Prepared models were then processed for hemodynamic analysis. The aneurysm model, stent, and coils were all imported into ANSYS ICEM CFD version 16.2 (ANSYS Inc., Canonsburg, Pennsylvania, United States) to mesh in a global mesh size of 0.16 mm. The surface mesh size of the stent (Enterprise 2 and Neuroform EZ) was set to 0.03 mm, and the mesh size of the coils was set to 0.1 mm (17). ANSYS CFX version 2019 (ANSYS Inc., Canonsburg, Pennsylvania, United States) was used to perform the CFD simulation on the basis of the Navier–Stokes equations. Blood flow was modeled as a laminar, incompressible, Newtonian fluid, whose viscosity and density were specified as 0.0035 kg/m·s and 1,056 kg/m3, respectively. The vascular wall was assumed as a rigid and no-slip boundary condition. The inlet flow rate was set to 4.6 mL/s, and the outlet pressure at every outlet branch was set to 0 (1, 18). In consideration of numeric stability, two cardiac cycle simulations were performed, and the final analysis results were obtained from the second cardiac cycle in this study.

## Hemodynamic measurements and analysis of distal intracranial arteries

### Location of distal vessel measurement

**Figure 1:**
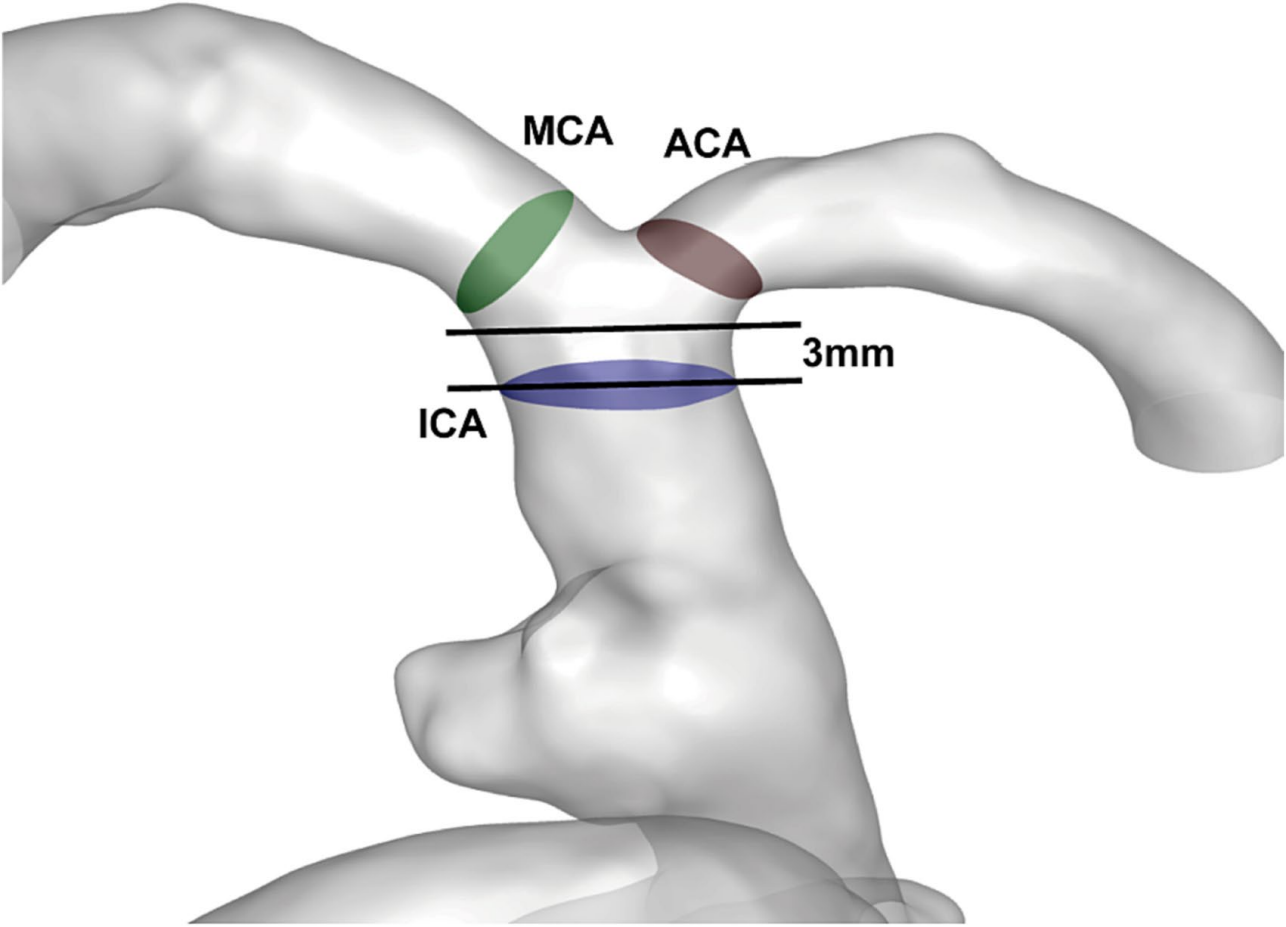
View of the measurement plane in the patient’s aneurysm and vascular model: the measurement planes were the test section of ICA (blue), the ACA (brown), and the MCA (green).

For both DICH and non-DICH patients, three interest regions within the distal arteries in the aneurysm were selected for hemodynamic measurements and analysis. These areas included the terminal segment of the ICA, as well as the origins of the ACA and MCA (1), as depicted in Figure 1. The detection plane for the ICA was defined as a point 3 mm proximal to its end. Similarly, the measurement sections for both the ACA and MCA were located 3 mm the point where they branch off from the ICA, aligned parallel to this bifurcation plane.

In patients with DICH, the responsible artery-either the ACA or the MCA-was identified using CT imaging (19). If DICH was located within the ACA’s territory, the ACA was designated as the DICH-related artery, and conversely, the MCA was considered unrelated. In cases where DICH manifested in the MCA’s region, the roles were reversed, with the MCA being the DICH-related artery and the ACA, the unrelated artery. Control group patients, without any incidents of DICH, were not subject to this classification. All angiographic evaluations and results were independently reviewed through a blinded process by two neuroradiologists, each with five years of experience in neurovascular imaging. A third neuroradiologist, with over eight years of clinical experience, adjudicated any discrepancies.

### The calculation and analysis of hemodynamic parameters

Initially, we quantified the maximum, mean, and minimum blood flow velocities within the ICA test section during peak systolic phase, both pre-and post-endovascular treatment, in the symptomatic DICH and control cohorts using ANSYS CFX version 2019. Furthermore, we assessed the rate of increase in blood flow velocity across all evaluated planes (ICA, ACA, and MCA) during peak systolic phase following SAC embolism, applying the formula: (post-procedure parameter-pre-procedure parameter)/pre-procedure parameter (1). This analysis aimed to ascertain if there were distinctive hemodynamic variations between the DICH-unrelated and related-arteries post-SAC embolism. For patients without DICH, we lacked comparable artery classifications, necessitating an analysis of hemodynamic shifts between between ACA and MCA. We employed the “imbalance index,” as delineated by Li et al. (1), to quantitatively gauge the disparity in flow alterations between the ACA and MCA post-treatment. This index represents the absolute difference in velocity change rates between the ACA and MCA, calculated as:

$\begin{array}{l} |\left(V_{\mathrm{POST}-MCA}-V_{PRE-MCA}\right)/V_{PRE-MCA}\\ -\left(V_{\mathrm{POST}-ACA}-V_{PRE-ACA}\right)/V_{PRE-ACA}| \end{array}$

A higher imbalance index value indicated a more pronounced asymmetry in flow distribution between the ACA and MCA following SAC, signifying a marked difference in flow velocity changes within these distal arteries. Here, “||” symbolizes the absolute value, with “V_Pre-ACA_,” “V_Post-ACA_,” “V_Pre-MCA_” and “V_Post-MCA_” denoting the respective velocities measured at the ACA and MCA before and after embolism.

### Statistical analysis

Propensity score matching (PSM) was employed to align patient cohorts with and without DICH. We meticulously analyzed clinical, demographic, and hemodynamic variables using the t-test, Mann–Whitney U-test, or chi-square test to address continuous data (both normally and non-normally distributed), and categorical data, respectively, for both groups. To assess hemodynamic discrepancies between DICH-unrelated and related arteries in DICH patients, Wilcoxon’s matched pairs signed-rank test was utilized. A *p*-value threshold of less than 0.05 was set to define statistical significance. All statistical evaluations was conducted using SPSS 22.0 software (SPSS Inc., Chicago, Illinois, United States).

## Results

### Aneurysmal and clinical characteristics in DICH group

In the DICH cohort of eight individuals, the gender distribution was three males and five females, with an average age of 57.38 ± 4.94 years, as detailed in Table 1. Each patient presented with unruptured aneurysms smaller than 10 mm. Six of these aneurysms were discovered during physical examination, while the remaining two patients reported headache symptoms. The onset of DICH varied: five patients experienced it more than 3 days post-procedure, two encountered this complication 2 days post-operation, and one patient manifested symptoms 24 h following treatment. The hematoma of four patients was located in the area supplied by ACA (ACA was the DICH related artery), and four patients were located in the area supplied by MCA (MCA was the DICH related artery). Post-DICH, two patients underwent craniotomy after DICH. Tragically, one patient died 3 days after the procedure, which included hematoma evacuation and bone flap decompression. The other patient entered a vegetative state subsequent to the same surgical intervention. For the remaining six patients, dual antiplatelet therapy was ceased upon DICH onset. They were then administered aspirin monotherapy starting one week after symptom emergence, continuing for six months. At the one-year follow-up, these patients showed near-complete resolution of DICH-related symptoms.

**Table 1:** Aneurysm characteristics and baseline clinical data in the DICH and control groups.

	DICH group (*n* = 8)	Control group (*n* = 16)	*p* value
Age, y	57.38 ± 4.94	53.63 ± 2.52	0.110
Female sex, *n* (%)	5 (62.5)	9 (56.25)	0.770
Hypertension, *n* (%)	6 (75)	13 (81.25)	0.883
Aneurysm size	5.66 ± 0.19	5.72 ± 0.15	0.690
Aneurysm neck	5.30 ± 0.17	5.58 ± 0.15	0.269
Dome-neck-ratio	1.05 ± 0.04	1.00 ± 0.02	0.726
Procedure duration, h	2.02 ± 0.05	1.963 ± 0.05	0.459
Iodinated contrast dosage, ml	92.96 ± 2.58	91.01 ± 1.61	0.510
Systolic pressure, mmHg	133.39 ± 1.76	131.94 ± 1.36	0.538
Diastolic pressure, mmHg	79.13 ± 0.90	77.06 ± 0.87	0.151
AA% inhibition	97.72 ± 1.22	94.66 ± 1.37	0.100
ADP% inhibition	61.45 ± 3.66	58.07 ± 2.27	0.421
Packing density	35.38 ± 0.68	34.73 ± 0.53	0.475
Kind of stent			0.681
Enterprise 2, *n* (%)	6 (75)	12 (75)	
Neuroform EZ, *n* (%)	2 (25)	4 (25)	
Stent diameter, mm	4.19 ± 0.10	4.13 ± 0.06	0.560
Stent length, mm	27.38 ± 1.28	25.25 ± 0.98	0.208
Angiographic result			0.723
Raymond grade 1, *n* (%)	7 (87.5)	14 (87.5)	
Raymond grade 2, *n* (%)	1 (12.5)	2 (12.5)	
Raymond grade 3, *n* (%)	0 (0)	0 (0)	

### Hemodynamic alteration of distal arteries in DICH group

DICH can be bifurcated into two distinct subtypes based on hemodynamics shifts in the DICH-related artery post-SAC embolism.

**Figure 2:**
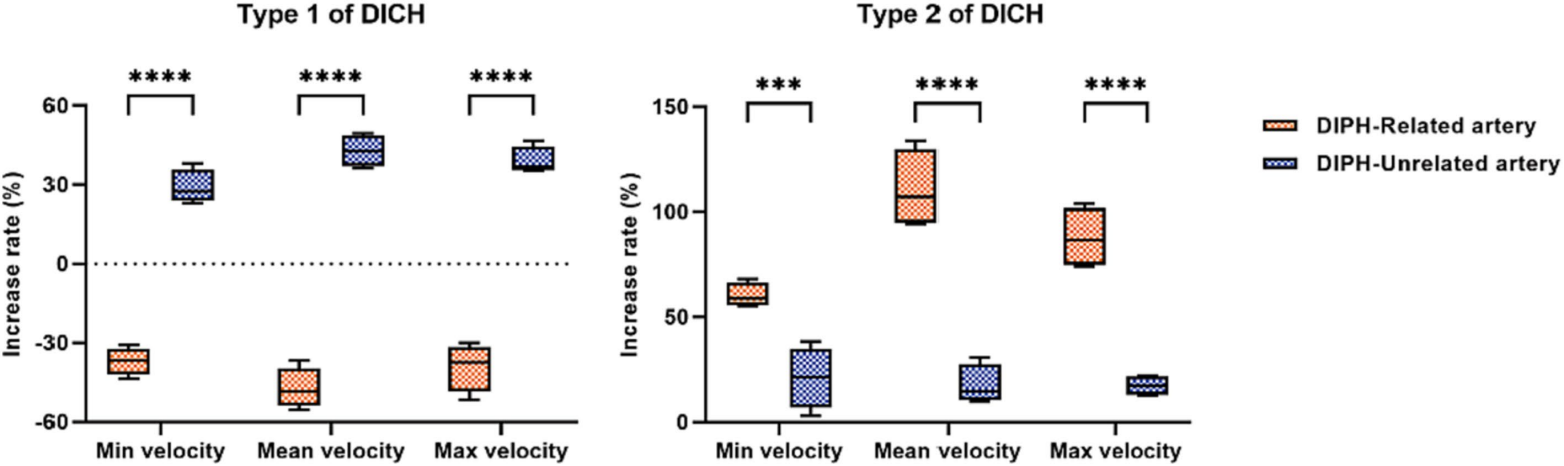
Comparison of the increase rate of velocity between DICH related and unrelated artery in both type 1 and type 2 DICH patients.

In type 1 DICH, a marked reduction in the postoperative minimum, mean and maximum rate of increase in velocity was observed in the DICH-related artery, in stark contrast to the DICH-unrelated artery, which were (−37.03 ± 2.62% vs. 29.10 ± 3.24%; *p* < 0.001), (−47.25 ± 3.88% vs. 42.85 ± 3.03%; *p* < 0.001), and (−39.08 ± 4.52% vs. 39.00 ± 2.64%; *p* < 0.001), respectively (Figure 2). Conversely, in type 2 DICH patients, notable increased were observed post-treatments, which were (60.15 ± 2.94% vs. 21.08 ± 7.27%; *p* = 0.002), (110.58 ± 9.42% vs. 17.60 ± 4.69%; *p* < 0.001), and (87.70 ± 7.22% vs. 17.25 ± 2.39%; *p* < 0.001), respectively (Figure 2). For DICH patients, SAC treatment notably accelerated blood flow velocity in the terminal segment of the ICA, as indicated in Table 2.

**Table 2:** Comparison of flow velocity at the test plane of ICA between pre-and post-procedure in patients with and without DICH.

	Min velocity			Mean velocity			Max velocity		
Group	Pre (m/s)	Post (m/s)	*p* value	Pre (m/s)	Post (m/s)	*p* value	Pre (m/s)	Post (m/s)	*p* value
DICH									
Type 1	0.073 ± 0.003	0.086 ± 0.003	0.025	0.383 ± 0.026	0.568 ± 0.020	0.001	0.475 ± 0.026	0.687 ± 0.016	<0.001
Type 2	0.077 ± 0.004	0.090 ± 0.006	0.135	0.386 ± 0.019	0.584 ± 0.036	0.003	0.472 ± 0.024	0.799 ± 0.040	<0.001
Control	0.076 ± 0.002	0.089 ± 0.002	<0.001	0.392 ± 0.008	0.589 ± 0.010	<0.001	0.478 ± 0.013	0.754 ± 0.004	<0.001

ICA: internal carotid artery.

### Distribution of distal arterial blood flow after SAC treatment in DICH group and control group

**Figure 3:**
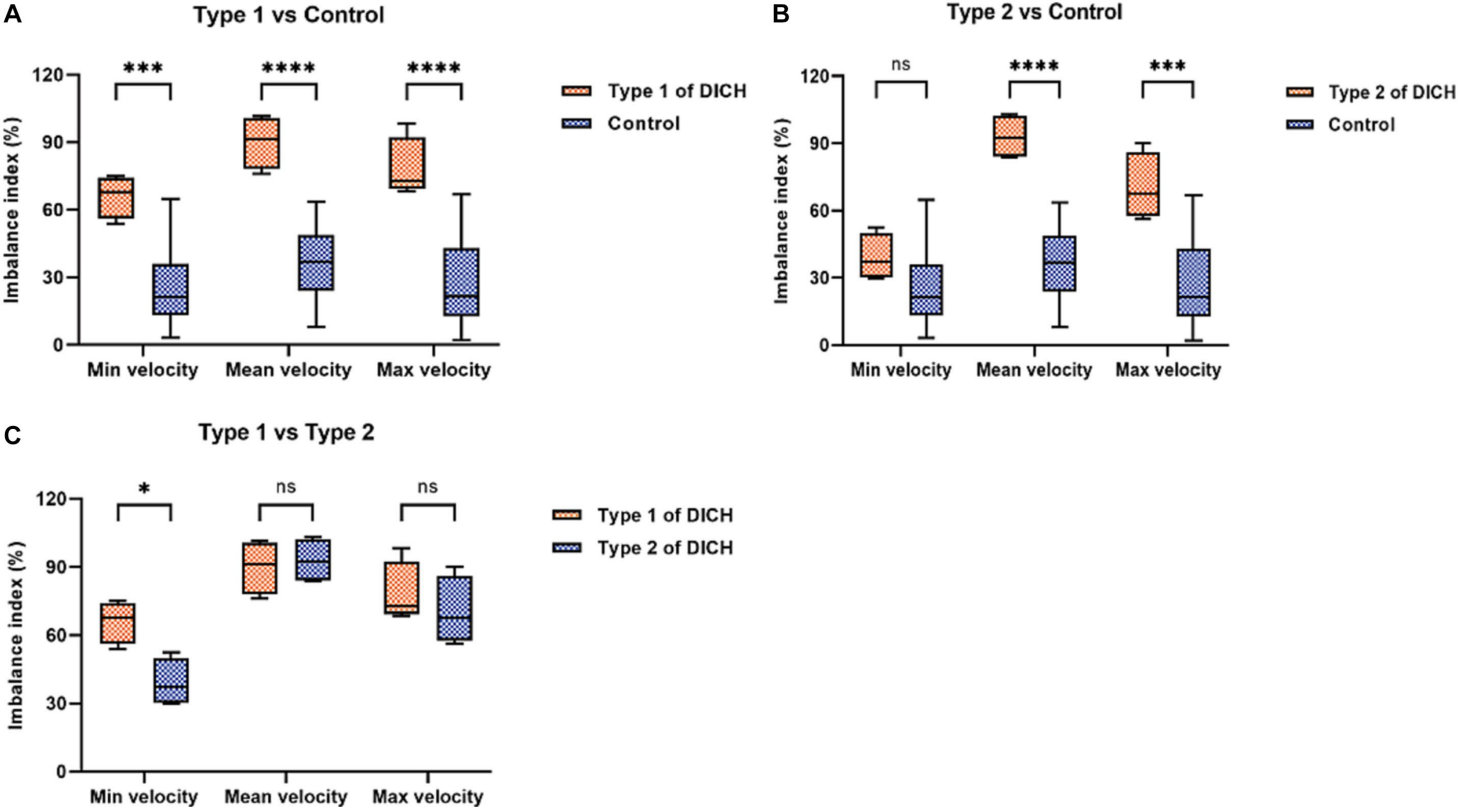
Comparison of the imbalance index of distal arteries in flow velocity between DICH group (both type 1 and type 2) and control group **(A,B)** and between type 1 and type 2 DICH patients **(C)**.

The baseline characteristics, encompassing clinical and aneurysmal profiles (Table 1) as well as pre-treatment hemodynamic (Table 3), were comparably matched between the two groups, exhibiting no significant statistical discrepancies. We assessed the blood flow distribution in distal arteries (ACA and MCA) before and after the procedure, as detailed in Tables 3, 4. Additionally, we calculated the imbalance indexes for distal artery flow in patients with and without DICH. Our findings revealed that type 1 DICH patients exhibited markedly higher imbalance index in minimum, mean and maximum flow velocities compared to those without DICH: 66.11 ± 4.77% vs. 25.19 ± 4.29% (*p* < 0.001), 90.08 ± 5.96% vs. 35.24 ± 4.39% (*p* < 0.001), and 78.07 ± 6.82% vs. 26.92 ± 4.91% (*p* < 0.001), respectively, as illustrated in Figure 3A. Similar trends were observed in the type 2 DICH group, with imbalance indexes of 39.09 ± 5.22% vs. 25.19 ± 4.29% (*p* = 0.143), 93.00 ± 5.10% vs. 35.24 ± 4.39% (*p* < 0.001), and 70.42 ± 7.55% vs. 26.92 ± 4.91% (*p* < 0.001), respectively, shown in Figure 3B. Notably, there was no significant difference in the imbalance index of mean and maximum flow velocities between type 1 and type 2 DICH patients: 90.08 ± 5.96% vs. 93.00 ± 5.10% (*p* = 0.722) and 78.07 ± 6.82% vs. 70.42 ± 7.55% (*p* = 0.480), respectively, as depicted in Figure 3C.

**Table 3:** Comparison of the velocity before SAC treatment in patients with and without DICH using Wilcoxon signed-rank test.

	ICA			ACA			MCA		
	DICH group type 1	Control group	*p* value	DICH group type 1	Control group	*p* value	DICH group type 1	Control group	*p* value
Min velocity (m/s)	0.073 ± 0.003	0.076 ± 0.002	0.493	0.060 ± 0.004	0.063 ± 0.002	0.502	0.055 ± 0.003	0.058 ± 0.002	0.490
Mean velocity (m/s)	0.383 ± 0.026	0.392 ± 0.008	0.669	0.455 ± 0.020	0.468 ± 0.008	0.491	0.332 ± 0.021	0.346 ± 0.007	0.447
Max velocity (m/s)	0.475 ± 0.026	0.478 ± 0.013	0.813	0.559 ± 0.020	0.549 ± 0.009	0.670	0.438 ± 0.026	0.455 ± 0.011	0.219

	DICH group type 2	Control group	*p* value	DICH group type 2	Control group	*p* value	DICH group type 2	Control group	*p* value
Min velocity (m/s)	0.077 ± 0.004	0.076 ± 0.002	0.721	0.061 ± 0.003	0.063 ± 0.002	0.602	0.056 ± 0.004	0.058 ± 0.002	0.678
Mean velocity (m/s)	0.386 ± 0.019	0.392 ± 0.008	0.850	0.458 ± 0.015	0.468 ± 0.008	0.559	0.339 ± 0.009	0.346 ± 0.007	0.681
Max velocity (m/s)	0.472 ± 0.024	0.478 ± 0.013	0.478	0.551 ± 0.028	0.549 ± 0.009	0.539	0.449 ± 0.020	0.455 ± 0.011	0.741

ACA: anterior cerebral artery; ICA: internal carotid artery; MCA: middle cerebral artery.

**Table 4:** Comparison of the increase rate of velocity in patients with and without DICH using Wilcoxon signed-rank test.

	ICA			ACA			MCA		
	DICH group type 1	Control group	*p* value	DICH group type 1	Control group	*p* value	DICH group type 1	Control group	*p* value
Min velocity (%)	17.93 ± 4.76	17.17 ± 1.87	0.865	−37.03 ± 2.62	15.48 ± 3.04	<0.001	29.10 ± 3.24	40.18 ± 3.37	0.089
Mean velocity (%)	49.85 ± 8.66	51.63 ± 4.39	0.858	−47.25 ± 3.88	15.96 ± 2.34	<0.001	42.85 ± 3.03	51.19 ± 3.73	0.295
Max velocity (%)	45.38 ± 5.46	59.26 ± 3.85	0.053	−39.08 ± 4.52	16.65 ± 1.40	<0.001	39.00 ± 2.64	43.57 ± 4.34	0.850

	DICH group type 2	Control group	*p* value	DICH group type 2	Control group	*p* value	DICH group type 2	Control group	*p* value
Min velocity (%)	16.43 ± 5.48	17.17 ± 1.87	0.872	21.08 ± 7.27	15.48 ± 3.04	0.436	60.15 ± 2.94	40.18 ± 3.37	0.009
Mean velocity (%)	51.05 ± 2.11	51.63 ± 4.39	0.949	17.60 ± 4.69	15.96 ± 2.34	0.758	110.60 ± 9.42	51.19 ± 3.73	<0.001
Max velocity (%)	69.98 ± 7.60	59.26 ± 3.85	0.228	17.25 ± 2.39	16.65 ± 1.40	0.846	87.70 ± 7.22	43.57 ± 4.34	0.007

ACA: anterior cerebral artery; ICA: internal carotid artery; MCA: middle cerebral artery.

**Figure 4:**
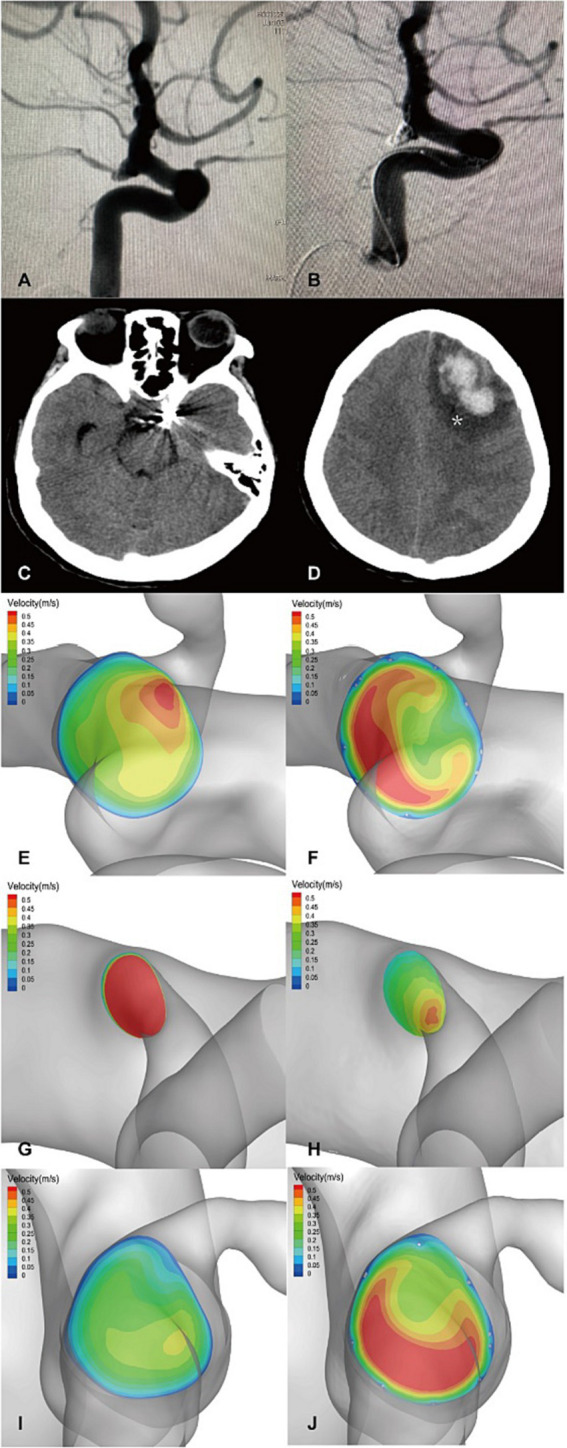
A posterior communicating segment aneurysm of left ICA in DICH of type 1 was treated with EP2 stent assisted coiling. **(A)** Preprocedural angiography of the aneurysm and **(B)** postprocedural immediate angiography revealed Raymond grade 2. **(C)** The coiled aneurysm was showed by CT scan at 24 h later after the treatment. **(D)** CT scan obtained 4 days post-procedure, revealing ipsilateral frontal lobe DICH with surrounding infarcted area (asterisk). A significant increase in velocity was observed on the terminal section of the ICA **(F)** compared to the preoperative results **(E)** by computer hemodynamic detection (Increase rate was 44.9%). The velocity of DICH related artery (ACA) decreased significantly after embolization with increase rate of-35.9% **(G,H)**, while the flow velocity of DICH unrelated artery (MCA) increased with increase rate of 35.7% **(I,J)**. The imbalance index of this patient was 71.6%.

**Figure 5:**
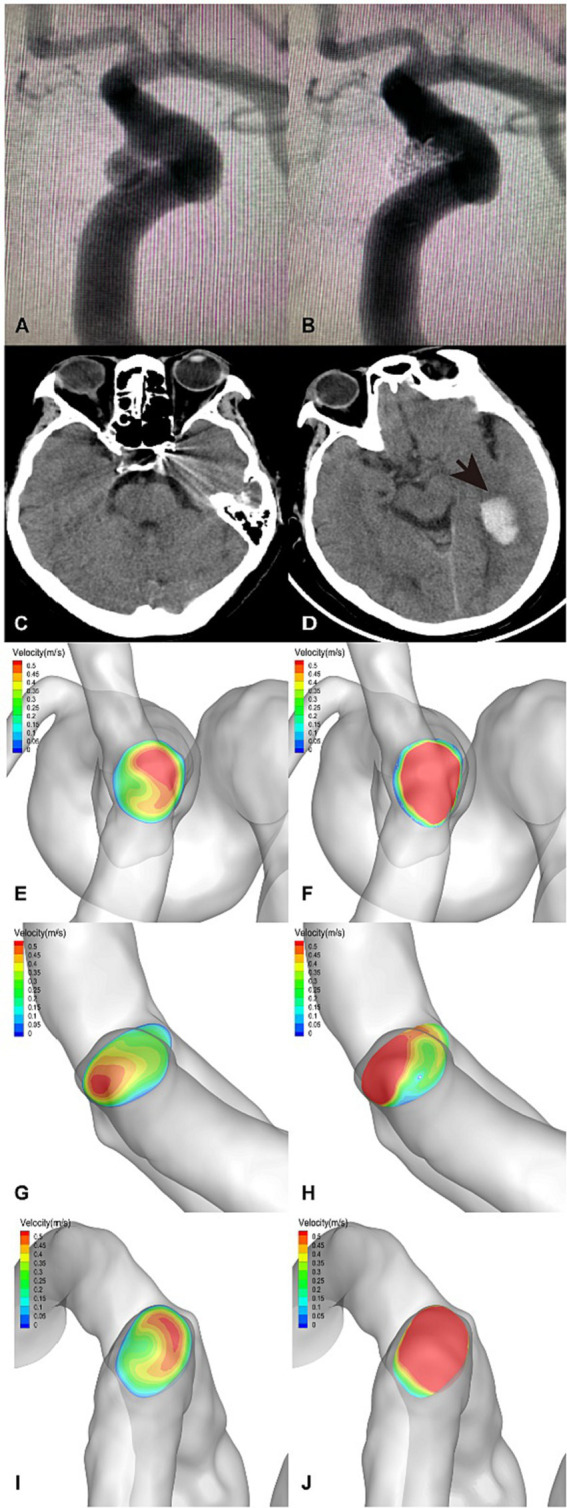
An ophthalmic segment aneurysm of the left ICA in DICH of type 2 was treated with EP2 stent assisted coiling. **(A)** Preprocedural angiography of the aneurysm and **(B)** postprocedural immediate angiography revealed Raymond grade 1. **(C)** The coiled aneurysm was showed by CT scan at 24 h later after the treatment. **(D)** CT scan obtained 3 days post-procedure, revealing ipsilateral temporal lobe DICH (arrow). A significant increase in velocity was observed on the terminal section of the ICA **(F)** compared to the preoperative results **(E)** by computer hemodynamic detection (Increase rate was 47.4%). The velocity of DICH related artery (MCA) increased significantly after embolization with an increase rate of 77.0% **(I,J)**, while the flow velocity of DICH unrelated artery (ACA) increased with increase rate of 20.7% **(G,H)**. The imbalance index of this patient was 56.3%.

**Figure 6:**
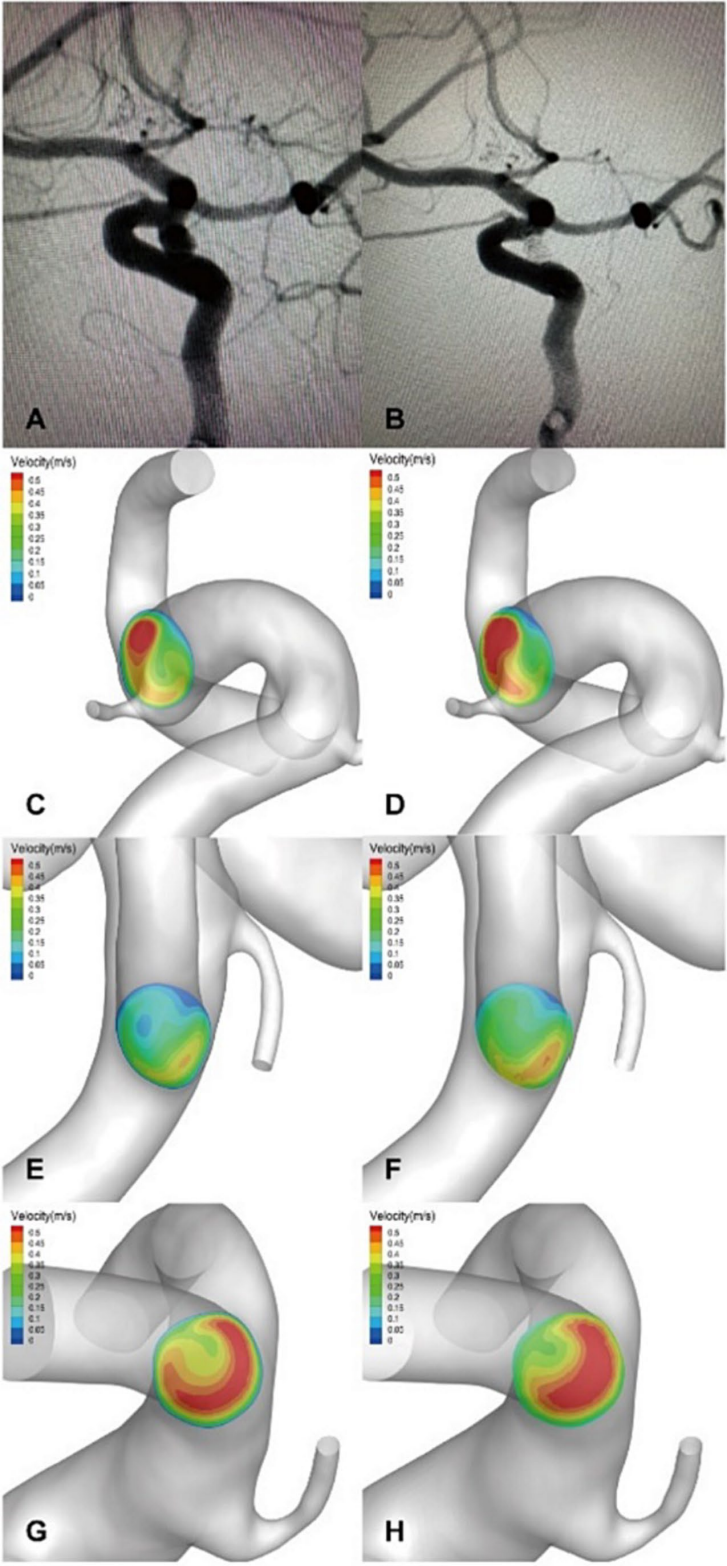
**(A,B)** An ophthalmic segment aneurysm in a left ICA without DICH in the control group was treated with EP2 stent assisted coiling, and a postprocedural immediate angiography revealed Raymond grade 1. A significant increase in velocity was observed on the terminal section of the ICA **(D)** compared to the preoperative results **(C)** by computer hemodynamic detection (Increase rate was 47.2%). The flow velocity of ACA **(E,F)** and MCA **(G,H)** increased significantly after embolization with increase rate of 22.5% and 41.3%, respectively. The imbalance index of this patient was 18.8%.

The flow velocity in the DICH-related artery (ACA) was significantly lower (as shown in Figures 4G,H), while the velocity in the DICH-unrelated artery (MCA) was higher (Figures 4I,J). Furthermore, remarkable infarcted areas were observed around the ipsilateral frontal lobe DICH on CT scans (Figure 4D), which were accompanied by a lower velocity in the DICH-related artery. Conversely, in type 2 DICH patients, the rate of velocity was notably higher in the DICH-related artery (MCA) (Figures 5I,J) compared to the DICH-unrelated artery (ACA) (Figures 5G,H). Additionally, flow velocity in all distal arteries (ACA and DICH unrelated MCA) was well-balanced and higher after SAC embolization in a patient from the control group (Figure 6).

## Discussion

DICH following endovascular treatment of intracranial aneurysms (IAs) is a critical yet unpredictable complication. While existing research primarily focuses on DICH post-FD treatment for IAs (4–25, 22, 20–23), the risk factors associated with DICH, particularly after SAC treatment for UIAs, remain under-explored. Our study highlights 1.5% incidence rate of DICH, typically manifesting 3.5 days post-procedure. Hemodynamic alterations allow us to categorize DICH into two distinct types. Type 1 DICH patients exhibited a significant lower in the postoperative velocity rate of the DICH-related artery compared to DICH-unrelated artery, whereas type 2 patients showed a notable higher.

Moreover, the imbalance index value serves as an indicator of the flow distribution variance in distal blood vessel. Our findings revealed that both type 1 and type 2 DICH patients had significantly higher imbalance index values compared to the control group. These data indicated that changes in velocity in the DICH-associated artery, leading to an augmented disparity in distal blood flow, could be a pivotal hemodynamic risk factor for DICH following SAC embolism.

Son et al. (2) reported that DICH in patients UIAs undergoing SAC embolization was linked to DATP, with an incidence rate mirroring our study’s findings at 1.5%. Hemorrhagic transformation of ischemic lesions has also been posited as a potential DICH cause (22, 26, 27). Nakae et al. (28) identified the average number of ischemic lesions post-pipeline embolization device (PED) placement as a bleeding predictor, noting a positive correlation with hemorrhagic transformations. The ischemic lesions might act as an initiating factor for DICH post-endovascular therapy, with DAPT potentially accelerating this process (2). Thus, we cautiously hypothesize that ischemic lesion hemorrhagic transformation significantly contributes to DICH in both FD and SAC embolism treatments.

Studies examining hemodynamics in DICH patients post-FD therapy suggest higher distal artery flow velocity post-flow diversion as a critical risk factor. Velat et al. (26) observed a case of DICH following complex intracranial aneurysm treatment with PED, attributing cerebral parenchymal hemorrhage to the redirection of blood flow by PED. Cruz et al. (22) reported DICH cases in anterior circulation aneurysms treated with PED, hypothesizing that PED implantation reduces aneurysmal segment compliance, alters the “Windkessel effect” (29, 30), and intensifies the blood pressure waveform in distal cerebral vessels, culminating in DICH. However, these theories lack robust clinical evidence. Brunozzi Denise et al. (5) used transcranial Doppler to track MCA flow changes post-PED, finding a more pronounced mean blood flow velocity in ipsilateral MCA in DICH patients. Studies indicated faster intracranial contrast transit times post-PED (14), with DICH patients showing greater reduction in MCA/ICA transit time ratio. These studies suggest that the altered hemodynamics of the intracranial distal vessels after PED treatment may play an important role in DICH. Li et al. (1) explored hemodynamic changes using computational fluid dynamics, proposing a link between DICH and imbalanced distal blood flow. This research differentiated between DICH-related and unrelated arteries, observing higher flow rate increases in DICH--related arteries post-treatment. Although these hemodynamic studies primarily focused on FD, we theorize similar changes in DICH post-SAC embolism. Our study indicates that the rate of increase in arterial velocity associated with DICH was higher in type 2 DICH patients than in controls. Additionally, the DICH group exhibited a significantly higher imbalance index. These hemodynamic shifts exacerbated the load on DICH-related arteries, disrupt the cerebral autoregulation (26, 31), and potentially lead to DICH.

Our research identified a distinct subtype of DICH, characterized by a significantly reduction in the velocity of the DICH-associated artery post-procedure. In this subtype, the imbalance index notably exceeded that of the control group. Hadad Sara’s research highlighted that FD implantation can reverse distal collateral blood flow (24), potentially leading to endothelial dysfunction and adversely affecting vascular wall integrity (32–34), thereby increasing the risk of DICH. Research has linked blood flow reversal to the activation of proinflammatory signals in endothelial cells (33, 35–37), predisposing the collateral vessels to hemorrhage. We hypothesize that in Type 1 DICH, flow reversal in the distal collateral arteries might trigger an inflammatory response, compromising vascular integrity. Additionally, the pronounced decrease in flow velocity of DICH-related arteries and heightened imbalance distal blood flow may induce cerebral parenchymal ischemia, culminating in hemorrhagic transformation, particularly under DAPT. In our study, CT imaging of a patient revealed a substantial infarct area adjacent to the ipsilateral frontal DICH (Figure 4D), alongside significant velocity reduction of in the DICH-associated artery. Collectively, these findings suggest that type 1 DICH may result from a confluence of multiple mechanisms.

This study is subject to several constraints. First of all, the inherent limitations of a non-randomized, retrospective design could influence the results. Secondly, the small sample size, attributable to the rarity of DICH post-SAC treatment, may affect the study’s findings. Future large-scale, multicenter, prospective studies are necessary to validate our hypotheses. Thirdly, multiple mechanisms, especially inflammatory responses, are likely involved in type 1 DICH., necessitating further investigation into the role of inflammatory factors in DICH. Finally, CFD hemodynamic analysis employed here has inherent limitations. Additional studies are required to evaluate the impact of assumptions such as Newtonian blood properties, laminar flow, boundary conditions, and elastic vessel walls on the hemodynamic outcomes.

## Conclusion

DICH following SCA treatment could be associated with hemodynamic alterations in the distal intracranial artery. Notably, the increase rate of velocity of DICH-related artery might either lower (as in type 1 DICH) or higher (as in type 2 DICH). The disproportionate flow distribution between ACA and MCA might serve as a potential predictive marker for these complications. Further research is required to substantiate these initial findings.

## Data availability statement

The raw data supporting the conclusions of this article will be made available by the authors, without undue reservation.

## Ethics statement

The studies involving humans were approved by the institutional medical ethics committee of Tongji Hospital, affiliated to Tongji Medical College of Huazhong University of Science and Technology (IRB ID: TJ20221004). The studies were conducted in accordance with the local legislation and institutional requirements. The participants provided their written informed consent to participate in this study.

## Author contributions

Z-BW: Conceptualization, Investigation, Methodology, Writing – original draft. X-YW: Conceptualization, Methodology, Writing – original draft. M-HZ: Data curation, Investigation, Writing – review & editing. Y-CL: Data curation, Investigation, Writing – review & editing. Z-ZM: Data curation, Investigation, Writing – review & editing. YZ: Data curation, Writing – review & editing. PL: Data curation, Software, Writing – review & editing. L-PG: Data curation, Software, Visualization, Writing – review & editing. J-PX: Data curation, Software, Writing – review & editing. H-QZ: Supervision, Writing – review & editing. KS: Supervision, Writing – review & editing. TL: Conceptualization, Project administration, Writing – review & editing. M-XZ: Conceptualization, Funding acquisition, Project administration, Writing – review & editing. AM: Writing - review & editing. XG: Writing - review & editing.
